# Non-contact radio frequency shielding and wave guiding by multi-folded transformation optics method

**DOI:** 10.1038/srep36846

**Published:** 2016-11-14

**Authors:** Hamza Ahmad Madni, Bin Zheng, Yihao Yang, Huaping Wang, Xianmin Zhang, Wenyan Yin, Erping Li, Hongsheng Chen

**Affiliations:** 1 Department of Electronic Engineering, Zhejiang University, Hangzhou 310027, China; 2 Institute of Marine Electronics Engineering, Zhejiang University, Hangzhou 310058, China

## Abstract

Compared with conventional radio frequency (RF) shielding methods in which the conductive coating material encloses the circuits design and the leakage problem occurs due to the gap in such conductive material, non-contact RF shielding at a distance is very promising but still impossible to achieve so far. In this paper, a multi-folded transformation optics method is proposed to design a non-contact device for RF shielding. This “open-shielded” device can shield any object at a distance from the electromagnetic waves at the operating frequency, while the object is still physically open to the outer space. Based on this, an open-carpet cloak is proposed and the functionality of the open-carpet cloak is demonstrated. Furthermore, we investigate a scheme of non-contact wave guiding to remotely control the propagation of surface waves over any obstacles. The flexibilities of such multi-folded transformation optics method demonstrate the powerfulness of the method in the design of novel remote devices with impressive new functionalities.

Radio frequency (RF) shielding^1^,^2^,^3^,^4^,^5^ plays an important role in semiconductor industries, which is generally used in circuits design to reduce the electromagnetic interference between different electronic components. It is also used in preventing the electromagnetic (EM) pollution that causes radiation poisoning or biological hazards^6^,^7^, such as shielded cap, brain coat, and lab coat etc. Furthermore, the employment of RF shielding is one of the most elegant and aesthetic term, which is also used in various shapes of invisibility cloaks^8^,^9^,^10^,^11^,^12^,^13^,^14^,^15^,^16^,^17^,^18^,^19^ to conceal the hidden objects from impinging with EM radiations.

The conventional RF shielding involves in the construction of enclosures by placing a barrier between the source of radiations and the area of protection, in order to minimize the transmission level of EM radiations that enter or leave the enclosed region. Various methods have been done to accomplish the RF shielding such as shield-attached method^20^ and RF shield method^21^ etc. However, in such conventional methods, the shielding region is not open to the outer world and even a pinhole gap in shielding material can permit the EM radiations to penetrate. Therefore, there is a need of “complete” enclosure and any part without shielding is considered as a leakage part. In addition, the coated shield requires additional space that is problematic for molding process and it is difficult to fully encapsulate the metal shield into the semi-conductor package. Hence, a need existed to provide a device and method to overcome the above limitations.

Differ from conventional method, in this paper, we investigated a new but simple scheme of non-contact RF shielding by applying transformation optics^22^,^23^,^24^,^25^,^26^ method that has potential to remotely create a shielding region from a distance where EM radiations cannot penetrate. This proposed idea is based on the compression of PEC boundary by applying multi-folded transformation optics method^27^,^28^, which leads to conversion of a closed shaped structure into an open shaped structure with the same functionality. We named such kind of device as an “open-shielded” device, which is independent of the position and shape of the shielded object and on one hand, there is no need of physically connection between the proposed device and the object. On the other hand, our proposed device contains gaps without leakage problem. The proposed open-shielded device is the perfect way of non-contact shielding with high anti-radiation performance and the merged PEC inside the proposed device gives excellent shielding effect. To further make our open-shielded device undetectable by radars, we extend our idea to make an open-carpet cloak. Furthermore, we achieve an object independent non-contact perfect wave guiding for surface wave. In the following, full wave finite element method is used to demonstrate the effectiveness of our proposed structures.

## Results and Discussion

**Figure 1:**
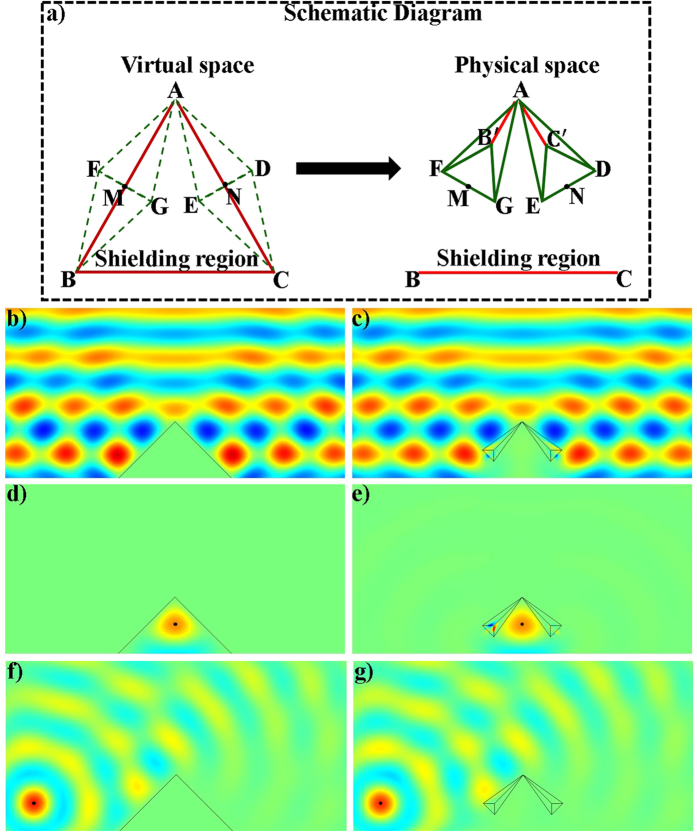
Scheme illustration of the proposed open-shielded device (**a**) and simulation results (**b**–**f**). (**a**) The red PEC triangle ABC in virtual space is divided into different regions denoted by green dashed lines. After transformation method applied on each triangle individually, an open-shielded device came into exist in physical space. (**b**–**g**) Simulation results for closed PEC shelter and the proposed open-shielded device with different source locations. (**b**,**c**) The electric field source is placed at the top of the PEC shelter and the proposed device, (**d**,**e**) A black dotted point source located inside the shielding region of the PEC shelter and the proposed device, (**f**,**g**) A black dotted point source is placed at the left side of the PEC shelter and the proposed device, respectively.

To start with, in the open-shielded device design, a closed PEC boundary structure i.e., a triangular shape colored in red, is shown in virtual space in Fig. 1(a). The PEC boundary is further divided into different triangular shapes, represented with green dashed lines, which are further transformed to make an open-shielded device in physical space (see Method Summary). From the transformation optics point of view, the whole structure in virtual space is optically equivalent to another structure described in physical space for the far-field viewers. Taking the left side of both virtual space and physical space as a reference as shown in Fig. 1(a), by applying compression and folding schemes, three distinguished regions came to exist. For detail, the regions Δ*ABF* and Δ*ABG* in virtual space are compressed into regions Δ*AB*′*F* and Δ*AB*′*G* in physical space respectively while region Δ*FGB* in virtual space is folded into region Δ*FGB*′ in physical space. Due to the structure symmetry, similar transformation method is applied in the right side. Hence, the original structure is mapped into the equivalent transformation medium and the proposed structure in physical space can be treated as an open-shielded device because its ability to create hidden region’s cross section area is much larger than its geometric size of PEC.

Simulation is performed in the frequency of 3.5 GHz. The detailed geometric and constitutive parameters are supported in Supplemental Information. Taking the left side as an example, the constitutive parameters are 
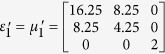
, 
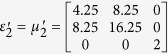
 and 
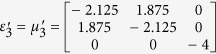
 corresponding to the regions Δ*AB*′*F*, Δ*AB*′*G* and Δ*FGB*′, respectively. In the following, we consider comparing the working performance of proposed device with the original PEC boundary with three different cases and the results are shown in Fig. 1(b–g). In the first case, the incident plane wave is propagating from top to bottom and the field distributions in both Fig. 1(b,c) are identical to each other. Therefore, the shielding region in the proposed device is undetectable for EM waves, as no waves can penetrate into that region. Another case, which can be observed in Fig. 1(d,e) that the waves inside the hidden region are also prohibited to leave that particular region, is verified by placing a point source inside of the shielding region. To further verify the all-angle shielding effect of the proposed device, a point source at the left side is used and the results of Fig. 1(e,f) validate the expected behavior of the proposed device.

In the above cases, the designed open-shielded device is realized by using homogeneous materials and there is no domain sharing among physical and effective PEC, either. These benefits bring more flexibility in designing the arbitrarily structures for shielding phenomenon. Although the size of PEC sheet in the compressed region is smaller than the size of PEC sheet before transformation, the functionality remains same. This is also the main advantage of our proposed device that the shielding region is physically open to the outer space and the EM radiations are not allowed to enter or leave that shielding region. Since the PEC can still be detected with this open-shielded device that raise a question: whether we are able to make that PEC undetectable, for example, make the PEC invisible to radar’s detection. As an answer, we further extend the open-shielded device’s concept into the carpet cloak to obtain an open-carpet cloak.

**Figure 2:**
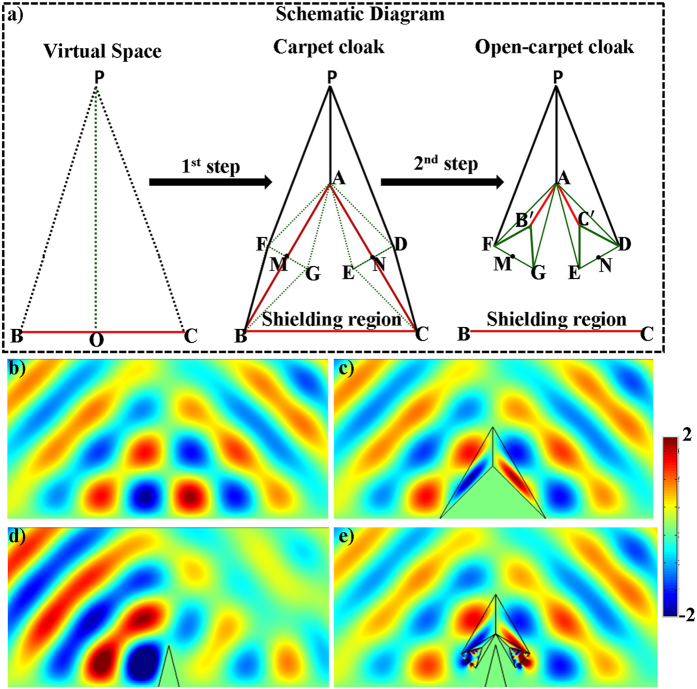
Schematic diagram and field distributions of open-carpet cloak. (**a**) Schematic diagram of open-carpet cloak showing the different steps. (**b**–**e**) The field distributions of (**b**) a flat reflection plane, (**c**) a traditional carpet cloak, (**d**) a PEC bump and (**e**) the bump with open-carpet cloak, respectively.

The schematic diagram of such open-carpet cloak design can be seen in Fig. 2(a), which contains two steps. At the first step, the virtual space is transformed into a conventional carpet cloak^29^,^30^ with two different regions such as Δ*PAB* and Δ*PAC*. The transformation equations and material parameters for such regions are obtained from ref. 29. Moreover, Δ*ABC* is the bump with PEC boundary and inside is free space. Thus, by taking advantage of this facility, in the next step, a similar transformation method is applied by recalling the Fig. 1(a) and in that way, an open-carpet cloak is designed. It should be noted that for regions Δ*AB*′*F* andΔ*AC*′*D*, which are compressed from virtual spaces with carpet cloak parameters, the material parameters of such regions are also obtained from that of carpet cloak (see Method Summary).

To verify the expected behavior of proposed open-carpet cloak, simulation is also performed and in this case, TE mode is applied with a Gaussian beam impinging the device with respect to *π*/4 at the frequency of 5 GHz. The detailed geometric and constitutive parameters are shown in Supplemental Information. Taking the left side as an example, the following constitutive parameters 
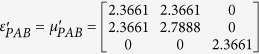
, 
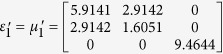
, 
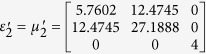
 and 
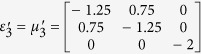
 are used for regions Δ*PAB*, Δ*AB*′*F*, Δ*AB*′*G*, and Δ*FGB*′, respectively. Hereafter, the simulation results are shown in Fig. 2(b–e). Figure 2(b) shows the field pattern of free space while Fig. 2(c) is a case of a traditional carpet cloak. Figure 2(d) is the result with bump only and Fig. 2(e) represents the remotely shielding of bump with the proposed open-carpet cloak. From simulation results, one can see that the field distribution of the open-carpet cloak is identical to the traditional one and independent of the “anti-object” concept^31^. Compared with the open-shielded device as discussed previously, in this case, the PEC is undetectable by radars and the device can hide any object from a certain distance. Hence, we demonstrated a simple method for designing of non-contact shielding devices with two different examples. In these cases, the object is free to move inside the shielding region and physically open to the outer space but the EM radiations at the operation frequency cannot enter or leave through the gap.

**Figure 3:**
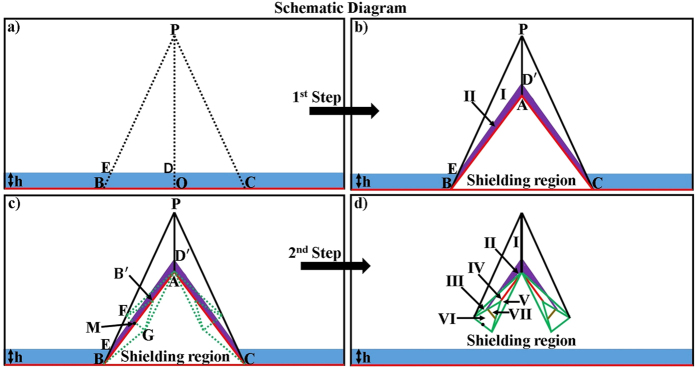
Schematic diagram of non-contact surface wave guiding. (**a**) A virtual space contained dielectric of *ε*_*dielectric*_ = 1 and *μ*_*dielectric*_ = −3 with the thickness of *h*, and then transformed it into a traditional carpet cloak^21^ for surface wave (**b**), while red lines show PEC. (**c**) Assume the (**b**) as a virtual space of our next proposed device with the transformation boundaries represented by green dashed lines. (**d**) After recalling the Fig. 1(a), a non-contact surface wave guiding for any object is designed with seven different regions.

Furthermore, this proposed method can also apply for realizing a non-contact wave guiding to control the propagation of surface wave from a certain distance. A surface wave transformation cloak has been proposed which operates with physically attachment to the dielectric substrate^32^,^33^. In the following, based on the concept of open-carpet cloak design with some minor change (see Method Summary), a non-contact surface wave guiding device is proposed, as shown in Fig. 3. For the first step, a transformation based surface wave cloak is obtained, with region I and region II in Fig. 3(b) transformed from air and dielectric in Fig. 3(a), respectively. In the second step, by recalling the Fig. 1(a), a similar transformation method is applied in Fig. 3(c) and the surface wave cloak turned into open cloak, as shown in Fig. 3(d). Again, it should be noted that in the second step of transformation, some regions in Fig. 3(d) are transformed from the virtual space with different parameters in Fig. 3(c), hence, the material parameters of such regions is also obtained. The detailed information is shown in Supplemental Information. Taking the left side as an example, the constitutive parameters 
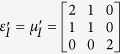
, 
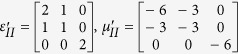
, 
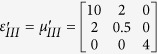
, 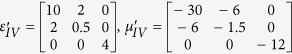, 
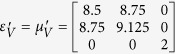
, 
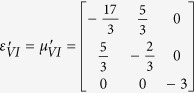
 and 
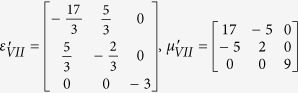
 are used for region I to region VII, respectively.

**Figure 4:**
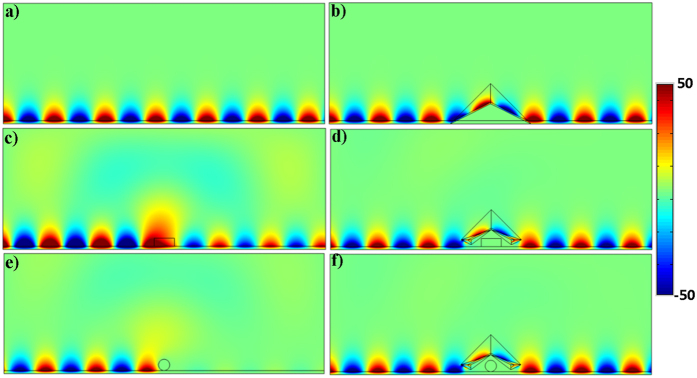
Simulation results of non-contact surface wave guiding. (**a**) A flat dielectric for surface wave. (**b**) Traditional carpet cloak for surface wave. (**c**) Dielectric bump of rectangular shape with *ε* = −6 and *μ* = 1 on the dielectric substrate caused disorder of surface wave and then proposed non-contact surface wave guiding device is used in (**d**) to remotely recover the surface wave over the bump. (**e**) A circular shape PEC bump on dielectric substrate then under the shelter of proposed device (**f**).

Simulations are performed to verify the proposed recipe for non-contact surface wave guiding device. The working frequency is 0.2 GHz and a dielectric substrate is used of *ε*_*dielectric*_ = 1 and *μ*_*dielectric*_ = −3, with the thickness of *h* = 0.04 *m*. The air/dielectric interface will support the propagation of magnetic surface wave. The detailed geometric and constitutive parameters are supported in Supplemental Information and Fig. 4 shows the simulated results for different cases. Figure 4(a) demonstrates the field pattern of the surface wave in free space and Fig. 4(b) is the case of surface wave propagating through a conventional transformation based surface wave cloak. In Fig. 4(c,d), a dielectric object with *ε* = −6, *μ* = 1 is placed at the top of the substrate, which will disorder the propagation of surface wave as shown in 4(c), while the field pattern is well recovered in 4(d). Furthermore, the proposed device is not limited to the object’s shape and material and this case is verified by placing a circular shaped PEC at the top of the substrate. The results are shown in Fig. 4(e,f), respectively. These simulation results are the bonus point to validate the proposed concept of this paper.

It should be noted that, the surface wave could jump from one PEC to another one through space due to the resonant nature of the negative index metamaterials. This is also the reason why the shielding device and the carpet cloak can work in an open way. The incident wave will resonate between the positive index material and negative index metamaterials to compensate each other. This resonance effect is also utilized to implement other novel devices such as electromagnetic gateways^34^ and superlens^35^ etc. If the wavelength is smaller or the space between the two PEC is very large, theoretically, this effect will still exist according to the wave theory because of the existence of both folding space and resonance. However, for numerical model, it requires finest mesh to demonstrate this wave behavior.

**Figure 5:**
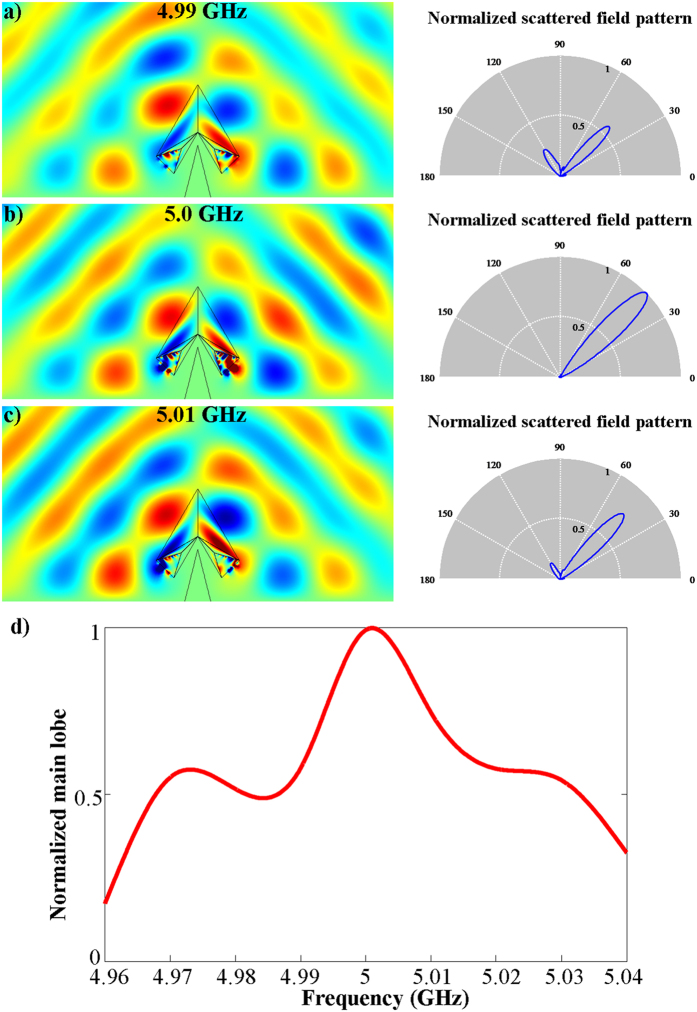
(**a**–**c**) Simulation results of open-carpet cloak and the normalized scattered field pattern with the dispersion model used in the negative index metamaterials at the frequencies of (**a**) 4.99 GHz, (**b**) 5 GHz, (**c**) 5.01 GHz. (**d**) The fitting curve of normalized main lobe for the different frequencies range from 4.96 GHz to 5.04 GHz.

The negative index metamaterials used in these open devices provokes several challenges: the key components are the losses and dispersions. In the following, we show how the dispersion affects the performance of our open devices. Taking the open carpet cloak as an example, for the dispersion models we use a Drude model for permittivity 
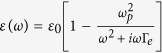
 and a Lorentz model for permeability 
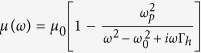
, where Γ_*e*_ and Γ_*h*_ refer to damping constants for both electric and magnetic field, respectively. For ease of discussion, we choose Γ_*e*_ = Γ_*h*_ = 0 and the resonant frequency *ω*_0_ is set to be equal to the plasma frequency *ω*_*p*_. At the working frequency of 5 GHz, different value of *ω*_*p*_ and *ω*_0_ are chosen separately to match the specific value calculated for the negative index metamaterials’ parameters. Simulations are also performed with different frequencies and the results are shown in Fig. 5. We can see from Fig. 5(b) that at the frequency of 5 GHz, the open device with ideal parameter can work well and the scattered field pattern has a main lobe at *π*/4 while the side lobes are smaller. When the dispersion modes are used and the frequency shifts a little, as shown in Fig. 5(a,c), the amplitude of the main lobe decreases while the side lobe increases. To show the bandwidth of our open device, we use the normalized main lobe amplitude to quantitatively represent the cloaking effect. Figure 5(d) represents the overall normalized main lobe amplitude for a set of different frequencies, which proves that dispersion will affect the shielding performance very much and that device can only operate in a small frequency band.

**Figure 6:**
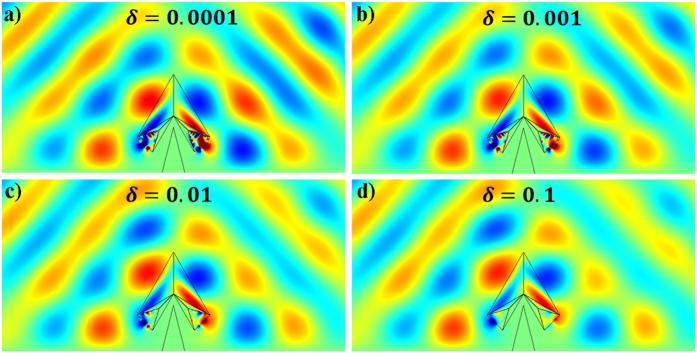
Simulation results of open-carpet cloak with different loss tangents used in the negative index metamaterials. (**a**) *δ* = 0.0001, (**b**) *δ* = 0.001, (**c**) *δ* = 0.01 and (**d**) *δ* = 0.1.

To further show how the loss will influence the performance of our open devices, we also make simulations with some losses in the negative index metamaterials. Taking the open carpet cloak as an example, the frequency is set to be 5 GHz and we use *ε* = *ε*_*r*_(1 + *iδ*) and *μ* = *μ*_*r*_(1 + *iδ*) for the negative values in the negative index metamaterials’ parameters, where *ε*_*r*_ and *μ*_*r*_ are the ideal parameters and *δ* is the loss tangent. In the simulations, we employ the loss tangent to be 0.0001, 0.001, 0.01 and 0.1, respectively. The total field distributions with different loss tangents are shown in Fig. 6. One can see that a big loss will destroy the shielding effect but the performance is still acceptable when the loss is relatively small.

## Conclusion

We proposed a non-contact method to remotely shield any object from EM waves and to guide surface waves above any obstacle by applying multi-folded transformation optics method. The proposed method is applicable for any shape of objects with any material parameters. With the help of multi-folded transformation technology, the sizes of open-devices can be designed in a much flexible way compare with the closed ones. Full-wave finite element method has been used to demonstrate the effectiveness of our method. A trade-off is that the constitutive parameters of the open devices will become increasing complicated and involves negative values, which leads to loss and dispersion. However, with the advantages of the non-contact properties, the proposed devices may find potential applications in microwave and optical engineering.

## Methods

The open-shielded device (Fig. 1(a)) is designed through a linear homogeneous transformation method for different regions. In details, taking the left side part as a reference, the region Δ*ABF* is compressed into region Δ*AB*′*F* with the following transformation function:


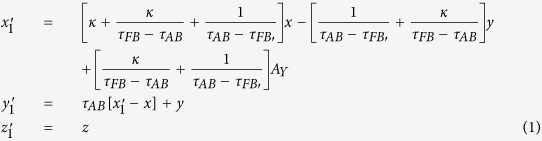


The compression of region Δ*ABG* into region Δ*AB*′*G* is similar with the transformation function:


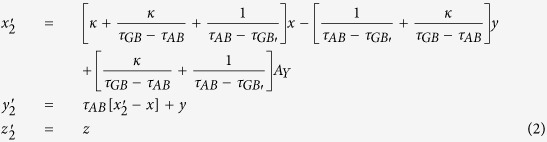


Hereafter, the region of Δ*FGB* is folded into Δ*FGB*′ with transformation function:


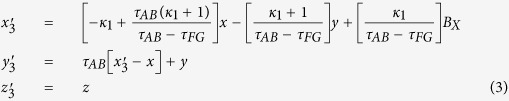


where, 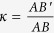 is the compression ratio, 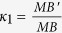 is the folding ratio, *τ*_*FB*_, *τ*_*FB*′_, *τ*_*AB*_, *τ*_*GB*_, *τ*_*GB*′_ and *τ*_*FG*_ are the slopes of lines *FB, FB*′, *AB, GB, GB*′ and *FG, A*_*Y*_ is the y-coordinate position of point *A* and *B*_*X*_ is the x-coordinate position of point *B*, respectively.

With the help of above mentioned transformation functions, the material parameters for each region are:


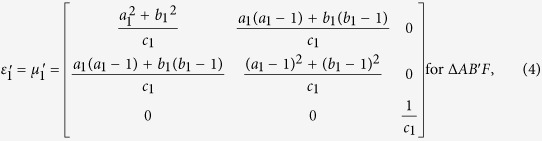



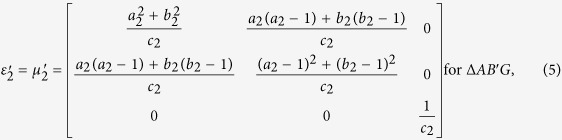



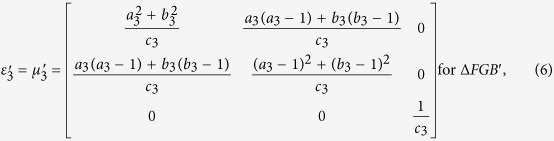


where 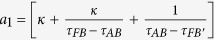, 
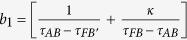
, *c*_1_ = −*a*_1_(*b*_1_ −1) + *b*_1_(*a*_1_ −1), 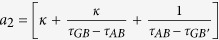, 
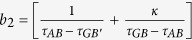
, *c*_2_ = −*a*_2_(*b*_2_ −1) + *b*_2_(*a*_2_ −1), 
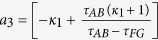
, 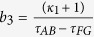, *c*_3_ = −*a*_3_(*b*_3_ − 1) + *b*_3_(*a*_3_ − 1), respectively.

For open-carpet cloak (Fig. 2(a)), the transformation contains two steps. At first step, a conventional carpet cloak is designed with the material parameters obtained from ref. 25. In the second step, by recalling the same method as shown in Fig. 1(a), an open-carpet cloak is designed. In which, Δ*ABF* and Δ*ABG* are further compressed into Δ*AB*′*F* and Δ*AB*′*G*, respectively while Δ*FGB* is folded into Δ*FGB*′ with same transformation function as mentioned in Eqs (1, 2, 3). Moreover, the designing methodology of non-contact surface wave guiding in Fig. 3 is similar to the open-carpet cloak design. In the first step, by applying the transformation functions from ref. 25, the material parameters for region I and region II can be obtained, which are transformed from the free space and the dielectric in virtual space, respectively. In the second step, by recalling the same method of Fig. 1(a), the material parameters for different regions can be obtained. In the Supplemental Information, a more detailed description of transformation as well as the settings of simulations is provided to show the material parameters of the proposed devices.

## Additional Information

**How to cite this article**: Madni, H. A. *et al*. Non-contact radio frequency shielding and wave guiding by multi-folded transformation optics method. *Sci. Rep.* **6**, 36846; doi: 10.1038/srep36846 (2016).

**Publisher’s note:** Springer Nature remains neutral with regard to jurisdictional claims in published maps and institutional affiliations.

## Supplementary Material

Supplementary Information
