# Supplementary material for: Non-contact radio frequency shielding and wave guiding by multi-folded transformation optics method
Source: Sci Rep. 2016 Nov 14;6:36846. doi: 10.1038/srep36846 (PMC5107927; doi:10.1038/srep36846)
Supplement: Supplementary Information [file srep36846-s1.pdf]

Non-contact radio frequency shielding and wave guiding by multi-folded transformation optics method

Hamza Ahmad Madni[1], Bin Zheng[1*], Yihao Yang[1], Huaping Wang[2*], Xianmin Zhang[1], Wenyan Yin[1], Erping Li[1], and Hongsheng Chen[1]

[1] Department of Electronic Engineering, Zhejiang University, Hangzhou 310027, China

[2] Institute of Marine Electronics Engineering, Zhejiang University, Hangzhou 310058, China

*Corresponding Authors: Bin Zheng (email): zhengbin@zju.edu.cn and Huaping Wang (email): hpwang@zju.edu.cn

I) Detailed transformation function of open-shielded device

In Fig. S1, the red lines show PEC while the green dashed lines are representing the transformation boundaries. It can be observed that ΔABF and ΔABG in virtual space is compressed into $\Delta AB'F$ and $\Delta AB'G$ respectively. Due to such compression, to make boundary condition matched and to overcome the discontinuity, we further applied folded transformation scheme in order to fold ΔFGB into $\Delta FGB'$.

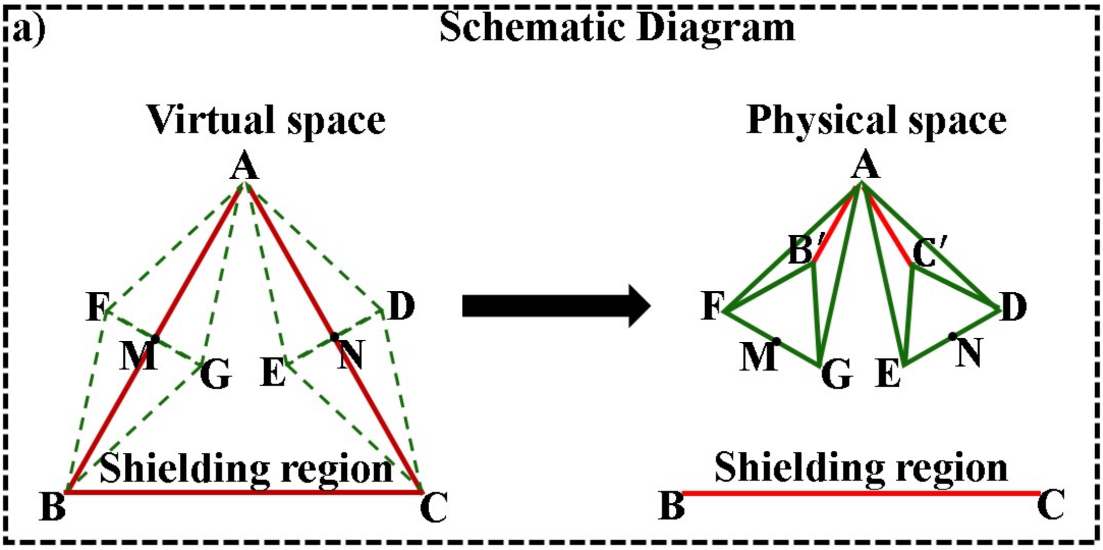

Fig. S1: Schematic diagram of open-shielded device

Before the simulation start, considering the left portion for both virtual and physical space of Fig. S1, we first assign the geometry coordinates in SI units as: $A(0,0.1)$, $B(-0.1,0)$, $F(-0.07,0.05)$, $G(-0.05,0.03)$ and $B'(0.05,0.05)$. In the following, we consider the transformation equations and the constitutive parameters for each region individually.

For ΔABF to $\Delta AB'F$:

$$\begin{aligned} x_1' &= 2.25x - 1.75y + 0.175 \\ y_1' &= 1.25x - 0.75y + 0.175 \\ z_1' &= z \end{aligned} \qquad \text{(S1)}$$

$$\varepsilon_1' = \mu_1' = \begin{bmatrix} 16.25 & 8.25 & 0 \\ 8.25 & 4.25 & 0 \\ 0 & 0 & 2 \end{bmatrix} \tag{S2}$$

For ΔABG **to** $\Delta AB'G$**:**

$$\begin{aligned} x_2' &= -0.75x + 1.25y - 0.125 \\ y_2' &= -1.75x + 2.25y - 0.125 \\ z_2' &= z \end{aligned} \tag{S3}$$

$$\varepsilon_2' = \mu_2' = \begin{bmatrix} 4.25 & 8.25 & 0 \\ 8.25 & 16.25 & 0 \\ 0 & 0 & 2 \end{bmatrix} \tag{S4}$$

For ΔFGB **to** $\Delta FGB'$**:**

$$\begin{aligned} x_3' &= 0.375x - 0.625y - 0.0125 \\ y_3' &= -0.625x + 0.375y - 0.0125 \\ z_3' &= z \end{aligned} \tag{S5}$$

$$\varepsilon_3' = \mu_3' = \begin{bmatrix} -2.125 & 1.875 & 0 \\ 1.875 & -2.125 & 0 \\ 0 & 0 & -4 \end{bmatrix} \tag{S6}$$

II) Detailed transformation function of Open-carpet cloak

Similarly, Fig. S2 represent the schematic diagram of proposed open-carpet cloak. For ease, we consider only the left side of device for demonstration, with the coordinate values of different points in SI units are $P(0, 0.0866)$, $B(-0.05, 0)$, $O(0, 0)$, $A(0, 0.05)$, $F(-0.0317, 0.0317)$, $G(-0.018, 0.018)$ and $B'(-0.0125, 0.0375)$.

In the first step, a virtual space with dashed lines are compressed into the solid lines such as ΔPOB is compressed into ΔPAB whereas, red lines show the PEC. The detailed transformation equations and constitutive parameters are obtained from ref. **1**. Such as:

$$x'_{PAB} = x$$
$$y'_{PAB} = \frac{\sqrt{3}-1}{\sqrt{3}} y + x + 0.05 \qquad \text{(S7)}$$
$$z'_{PAB} = z$$

$$\varepsilon'_{PAB} = \mu'_{PAB} = \begin{bmatrix} 2.3661 & 2.3661 & 0 \\ 2.3661 & 2.7888 & 0 \\ 0 & 0 & 2.3661 \end{bmatrix} \qquad \text{(S8)}$$

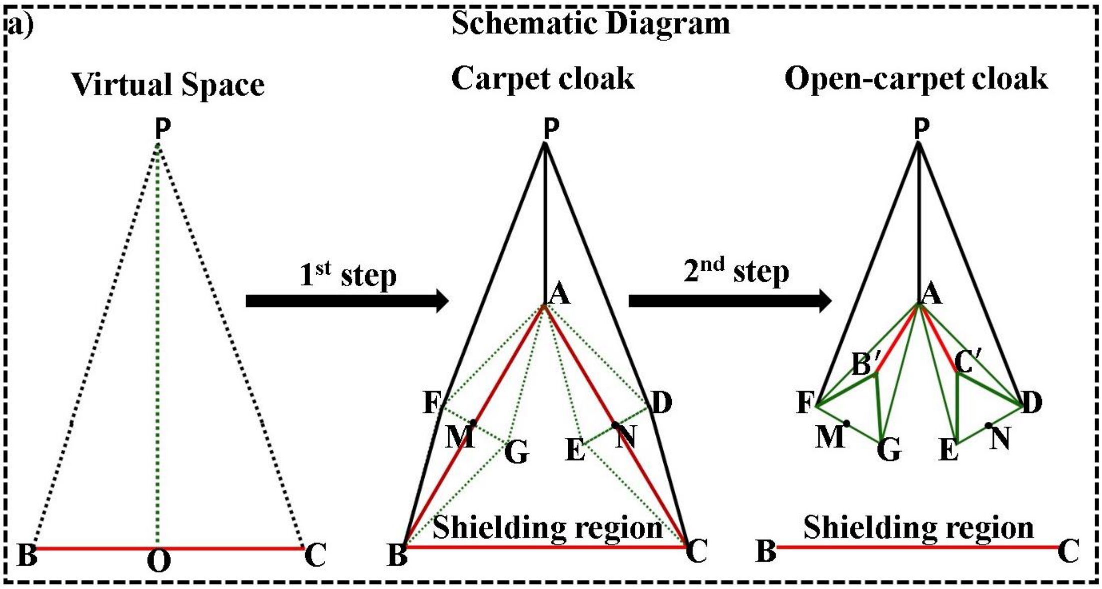

Figure S2: Schematic diagram of proposed open-carpet cloak

In the second step, traditional carpet cloak is assumed as a virtual space of our next proposed device while the green dashed lines show the transformation boundaries. After recalling the method shown in Fig. S1, open carpet cloak is achieved. Fore illustration, ΔABF and ΔABG are further compressed into $\Delta AB'F$ and $\Delta AB'G$ respectively. It should be noticed that the material parameters for $\Delta AB'F$ is obtained from the virtual space with parameters as shown in Eq. (S8).

For ΔABF **to** $\Delta AB'F$ **:** The transformation equations and material parameters are given as:

$$\begin{aligned} x_1' &= \frac{1085}{536}x - \frac{951}{536}y + \frac{951}{10720} \\ y_1' &= \frac{549}{536}x - \frac{415}{536}y + \frac{951}{10720} \\ z_1' &= z \end{aligned} \tag{S9}$$

$$\varepsilon_1' = \mu_1' = \begin{bmatrix} 5.9141 & 2.9142 & 0 \\ 2.9142 & 1.6051 & 0 \\ 0 & 0 & 9.4644 \end{bmatrix} \tag{S10}$$

For ΔABG to $\Delta AB'G$: The transformation equations and material parameters are given as:

$$\begin{aligned} x_2' &= -\frac{5}{7}x + \frac{27}{28}y - \frac{27}{560} \\ y_2' &= -\frac{12}{7}x + \frac{55}{28}y - \frac{27}{560} \\ z_2' &= z \end{aligned} \tag{S11}$$

$$\varepsilon_2' = \mu_2' = \begin{bmatrix} 5.7602 & 12.4745 & 0 \\ 12.4745 & 27.1888 & 0 \\ 0 & 0 & 4 \end{bmatrix} \tag{S12}$$

For ΔFGB to $\Delta FGB'$: The transformation equations and material parameters are given as:

$$\begin{aligned} x_3' &= 0.25x - 0.75y \\ y_3' &= -0.75x + 0.25y \\ z_3' &= z \end{aligned} \tag{S13}$$

$$\varepsilon_3' = \mu_3' = \begin{bmatrix} -1.25 & 0.75 & 0 \\ 0.75 & -1.25 & 0 \\ 0 & 0 & -2 \end{bmatrix} \tag{S14}$$

III) Detailed transformation function of Non-contact surface wave guiding

In the following, we consider the non-contact wave guiding for surface waves with the schematic diagram given in Fig. S3. It contains two steps and at first step, a) virtual space contains dielectric in blue shaded color and the red line shows PEC. The

black dashed lines show the transformation boundary similar as that of open-carpet cloak (Fig. S2). Furthermore, the coordinate positions of each point in the first step of the device in SI units are $P(0,0.5)$, $O(0,0)$, $C(0.5,0)$, $A(0,0.25)$, $B(-0.5,0)$ and $E(-0.46,0.04)$.

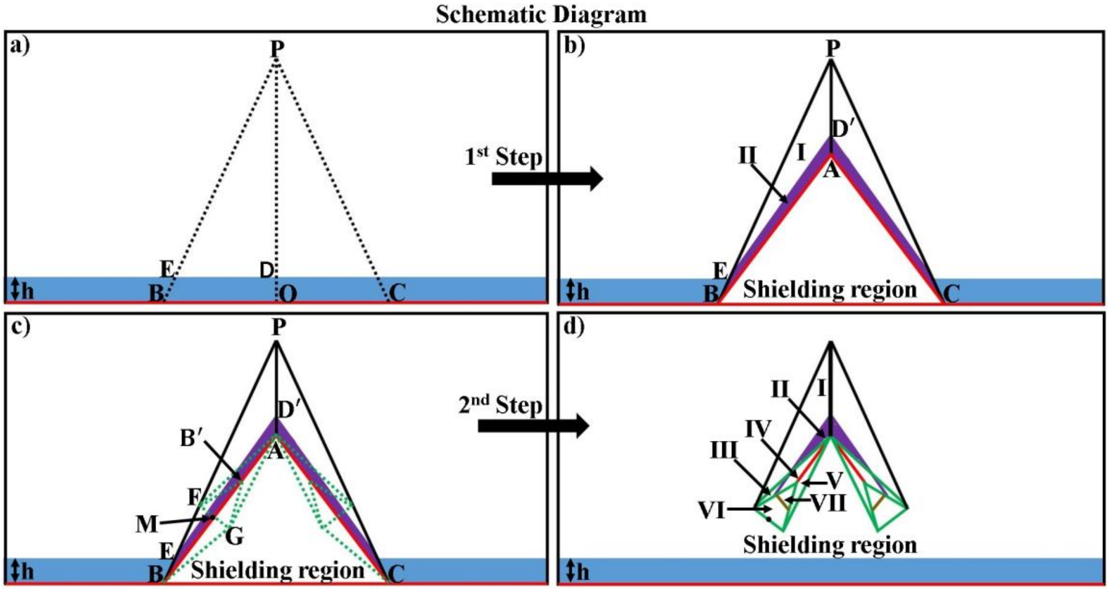

Figure S3: Schematic diagram of proposed open-carpet cloak

Before moving to second step, we first find the transformation equations and material parameters of each regions of first step.

First step: In Fig. S3 (a), ΔPOB is transformed into ΔPAB with the transformation equations, given as:

$$\begin{aligned} x'_{PAB} &= x \\ y'_{PAB} &= \frac{1}{2}y + \frac{1}{2}x + 0.25 \\ z'_{PAB} &= z \end{aligned} \tag{S15}$$

So, the material parameters for region I will become as:

$$\varepsilon'_I = \mu'_I = \begin{bmatrix} 2 & 1 & 0 \\ 1 & 1 & 0 \\ 0 & 0 & 2 \end{bmatrix} \tag{S16}$$

It can be observed that during the compression of ΔPOB to ΔPAB, the point D also shifted to D'. If the original coordinate position of D is (0, 0.04) then, by placing these values in Eq. (S15), the coordinate position of D' is obtained as (0, 0.27), while point E remains unchanged. In fact, the transformation of D to D' indicates the region II as a carpet cloak region with dielectric parameters because the transformation ratio is similar as that Eq. (S15). If dielectric substrate is used of $\varepsilon_{dielectric} = 1$, $\mu_{dielectric} = -3$ with the thickness of $h = 0.04m$, then the material parameters for region II will become as:

$$\varepsilon'_{II} = \begin{bmatrix} 2 & 1 & 0 \\ 1 & 1 & 0 \\ 0 & 0 & 2 \end{bmatrix}, \mu'_{II} = \begin{bmatrix} -6 & -3 & 0 \\ -3 & -3 & 0 \\ 0 & 0 & -6 \end{bmatrix} \tag{S17}$$

Second step: In the second step, we assume that device as a virtual space of our next proposed non-contact surface wave guiding, can be seen in Fig. S3(c). Due to symmetrical structure of the device, here we consider only the left portion of the device and the position coordinates of each points are given is SI units such as: $F = (-0.375, 0.125)$, $G = (-0.25, 0.0625)$, $B' = (-0.25, 0.125)$. After recalling the Fig. S1's method, a non-contact surface wave-guiding device with seven different regions has been designed, which can be seen in Fig. S3 (d).

It should be noticed that ΔABF contains a portion of both region I and II and after compression to $\Delta AB'F$, region III and IV came to exist respectively. While, ΔABG contains air, and after compression to $\Delta AB'G$, region V came to exist. Due to the compression, we need complementary (folding) region to overcome the discontinuity. In this way, ΔFGB is folded into $\Delta FGB'$ with some extra information. As physical space contains both air and dielectric, so the resultant folding region must

be composed of two different regions like region VI and VII, respectively. In the following, the transformation equations and material parameters for each region are discussed.

For ΔABF **to** $\Delta AB'F$ **:** The transformation equations and material parameters are given as:

$$\begin{aligned} x_1' &= 2x - 3y + 0.75 \\ y_1' &= \frac{1}{2}x - \frac{1}{2}y + 0.375 \\ z_1' &= z \end{aligned} \tag{S18}$$

The material parameters for region III and IV can be determined as:

$$\varepsilon_{III}' = \mu_{III}' = \begin{bmatrix} 10 & 2 & 0 \\ 2 & 0.5 & 0 \\ 0 & 0 & 4 \end{bmatrix} \tag{S19}$$

$$\varepsilon_{IV}' = \begin{bmatrix} 10 & 2 & 0 \\ 2 & 0.5 & 0 \\ 0 & 0 & 4 \end{bmatrix}, \mu_{IV}' = \begin{bmatrix} -30 & -6 & 0 \\ -6 & -1.5 & 0 \\ 0 & 0 & -12 \end{bmatrix} \tag{S20}$$

For ΔABG **to** $\Delta AB'G$ **:** The transformation equations and material parameters are given as:

$$\begin{aligned} x_2' &= -0.5x + 2y - 0.5 \\ y_2' &= -0.75x + 2y - 0.25 \\ z_2' &= z \end{aligned} \tag{S21}$$

$$\varepsilon_{V}' = \mu_{V}' = \begin{bmatrix} 8.5 & 8.75 & 0 \\ 8.75 & 9.125 & 0 \\ 0 & 0 & 2 \end{bmatrix} \tag{S22}$$

For ΔFGB **to** $\Delta FGB'$ **:** The transformation equations and material parameters are given as:

$$x_3' = \frac{1}{3}x - \frac{4}{3}y - \frac{1}{12}$$
$$y_3' = -\frac{1}{3}x + \frac{1}{3}y - \frac{1}{24} \qquad \text{(S23)}$$
$$z_3' = z$$

For air portion: $$\varepsilon_{VI}' = \mu_{VI}' = \begin{bmatrix} -\frac{17}{3} & \frac{5}{3} & 0 \\ \frac{5}{3} & -\frac{2}{3} & 0 \\ 0 & 0 & -3 \end{bmatrix} \qquad \text{(S24)}$$

For dielectric portion: $$\varepsilon_{VII}' = \begin{bmatrix} -\frac{17}{3} & \frac{5}{3} & 0 \\ \frac{5}{3} & -\frac{2}{3} & 0 \\ 0 & 0 & -3 \end{bmatrix}, \mu_{VII}' = \begin{bmatrix} 17 & -5 & 0 \\ -5 & 2 & 0 \\ 0 & 0 & 9 \end{bmatrix} \qquad \text{(S25)}$$

Reference

[1] Xi, S., Chen, H., Wu, B-I., & Kong, J. A. One-directional perfect cloak created with homogeneous material. IEEE Microw. Wirel. Co. **19**, 131-133 (2009).
